# Exogenous HGF Bypasses the Effects of ErbB Inhibition on Tumor Cell Viability in Medulloblastoma Cell Lines

**DOI:** 10.1371/journal.pone.0141381

**Published:** 2015-10-23

**Authors:** Walderik W. Zomerman, Sabine L. A. Plasschaert, Sander H. Diks, Harm-Jan Lourens, Tiny Meeuwsen-de Boer, Eelco W. Hoving, Wilfred F. A. den Dunnen, Eveline S. J. M. de Bont

**Affiliations:** 1 Department of Pediatric Oncology/Hematology, Beatrix Children’s Hospital, University Medical Center Groningen, Groningen, The Netherlands; 2 Princess Máxima Center for Pediatric Oncology, Utrecht, the Netherlands; 3 Department of Neurosurgery, University Medical Center Groningen, Groningen, The Netherlands; 4 Department of Pathology and Medical Biology, University Medical Center Groningen, Groningen, The Netherlands; Seoul National University, REPUBLIC OF KOREA

## Abstract

Recent clinical trials investigating receptor tyrosine kinase (RTK) inhibitors showed a limited clinical response in medulloblastoma. The present study investigated the role of micro-environmental growth factors expressed in the brain, such as HGF and EGF, in relation to the effects of hepatocyte growth factor receptor (MET) and epidermal growth factor receptor family (ErbB1-4) inhibition in medulloblastoma cell lines. Medulloblastoma cell lines were treated with tyrosine kinase inhibitors crizotinib or canertinib, targeting MET and ErbB1-4, respectively. Upon treatment, cells were stimulated with VEGF-A, PDGF-AB, HGF, FGF-2 or EGF. Subsequently, we measured cell viability and expression levels of growth factors and downstream signaling proteins. Addition of HGF or EGF phosphorylated MET or EGFR, respectively, and demonstrated phosphorylation of Akt and ERK1/2 as well as increased tumor cell viability. Crizotinib and canertinib both inhibited cell viability and phosphorylation of Akt and ERK1/2. Specifically targeting MET using shRNA’s resulted in decreased cell viability. Interestingly, addition of HGF to canertinib significantly enhanced cell viability as well as phosphorylation of Akt and ERK1/2. The HGF-induced bypass of canertinib was reversed by addition of crizotinib. HGF protein was hardly released by medulloblastoma cells itself. Addition of canertinib did not affect RTK cell surface or growth factor expression levels. This manuscript points to the bypassing capacity of exogenous HGF in medulloblastoma cell lines. It might be of great interest to anticipate on these results in developing novel clinical trials with a combination of MET and EGFR inhibitors in medulloblastoma.

## Introduction

Medulloblastoma is the most common malignant pediatric brain tumor and accounts for approximately 15–20% of all pediatric brain tumors[1]. The 5-year event free survival of medulloblastoma patients has increased to approximately 80% in the average-risk group and 50–60% in the high-risk group. Treatment consists of a combination of neurosurgery, cranio-spinal radiotherapy and chemotherapy, often resulting in long-term neurological and psychological side effects in the majority of survivors[2–5]. Specifically targeting the tumor cells with novel therapies might improve survival as well as decrease the long-term side effects.

Transcriptional profiling studies in medulloblastoma identified four distinct molecular subgroups based upon clustering of genes that activate key signaling pathways involved in tumor cell survival and proliferation: Wingless (Wnt)-subgroup (~10%), Sonic Hedgehog (SHH)-subgroup (~30%), Group 3 (~25%) and Group 4 (~35%)[6,7]. These subgroups have distinct transcriptional and genetic profiles, patient demographics and clinical behavior. In the activation of signaling pathways the tumor microenvironment also plays an important role. Various receptor tyrosine kinases (RTK’s) are expressed in medulloblastoma, including vascular endothelial growth factor receptor-2 (VEGFR-2), platelet-derived growth factor receptor α (PDGFRα), hepatocyte growth factor receptor (MET) and epidermal growth factor receptor 2 (ErbB2)[8]. Important growth factors present in the central nervous system include VEGF, PDGF, HGF, FGF and EGF[9–13]. These growth factors can activate specific RTK’s on the tumor cell surface. Phosphorylation of RTK’s generates a cascade of signals through common critical downstream signaling pathways involved in cell survival and proliferation, e.g. MAPK/ERK and PI3K/Akt pathways[8]. With kinome profiling we previously observed kinase-induced phosphorylation of peptide sequences derived from different RTK’s in medulloblastoma patient samples. These RTK’s include MET and ErbB2[14]. High expression levels of MET and ErbB2 are correlated with poor clinical outcome in medulloblastoma patients[15,16]. ErbB2 is unable to bind any known ligand and needs heterodimerization with other ErbB receptor family members (EGFR, ErbB3, ErbB4) for activation of its intracellular kinase domain. Therefore, MET and all ErbB family receptors might be interesting targets for the treatment of medulloblastoma patients with RTK inhibitors.

Currently, numerous RTK inhibitors have been developed ready for use in pediatric clinical trials. MET inhibitor crizotinib is currently being assessed for its anti-tumor activity in a pediatric clinical trial, including medulloblastoma (NCT00939770). In addition, ErbB TK inhibitors (lapatinib and erlotinib) have already been used in phase I/II clinical trials analyzing their anti-tumor activity in children (NCT00095940; NCT00077454). ErbB TK inhibitors were well tolerated, but more importantly, showed a limited clinical response in medulloblastoma patients[17,18]. A potential mechanism of tumor resistance against RTK inhibitors was found in non small-cell lung cancer (NSCLC) and HER2-positive breast cancer, where tumors became resistant to EGFR inhibition as a consequence of MET gene amplification[19,20]. Furthermore, various mutated or amplified cancer cell lines with kinase-dependency were able to bypass the growth-inhibitory effects of specific RTK inhibitors after addition of growth factors usually secreted by the tumor microenvironment[21–23]. Although various growth factors are present in the central nervous system, the role of microenvironmental growth factors in relation to the effects of specific RTK inhibitors in medulloblastoma needs to be elucidated further.

The present study examines the role of microenvironmental growth factors in relation to the effects of RTK inhibitors crizotinib and canertinib on tumor cell viability and downstream signaling in medulloblastoma cell lines.

## Materials and Methods

### Cell cultures and treatments

Human medulloblastoma cell line DAOY was purchased from the American Type Culture Collection (ATCC), human medulloblastoma cell lines RES256, UW402, UW426 and UW473 were a kind gift of Dr Michael S. Bobola, (Seattle Children’s Hospital Research Institute)[24]. Medulloblastoma cell lines were cultured in DMEM/F12 growth medium at 37°C in a humidified atmosphere of 5% CO2. Growth medium was supplemented with 5% fetal calf serum (FCS) (GIBCO) 100 units ml-1 penicillin and 100μg ml-1 streptomycin (GIBCO). Cells were treated with RTK inhibitors crizotinib, a dual inhibitor of MET and ALK, and canertinib, targeting the ErbB receptor family (EGFR, ErbB2 and ErbB4) (LC laboratories, Woburn, MA, USA)[25,26]. Cells were stimulated with growth factors, including VEGF-A, PDGF-AB, HGF, FGF-2 and EGF (up to 100ng/ml, Life Technologies).

### Flow cytometry analyses

Cells were treated with or without crizotinib or canertinib for one day. Cells were harvested by trypsinization, washed and blocked by PBS 1% Bovine Serum Albumin (BSA) (Sigma, St Louis, MO, USA) and stained with anti-VEGFR-1 (#MAB321, Sigma Aldrich), anti-VEGFR-2 (#V9134, Sigma Aldrich), anti-VEGFR-3/APC (#FAB3492, R&D systems, Minneapolis, MN, USA), anti-PDGFRα/biotin (#13-1401-80, eBiosciences, Vienna, Austria) and anti-PDGFRβ/PE (#558821, BD Biosciences), anti-MET/FITC (#FAB3582, R&D systems), anti-FGFR-1 (#91740, Cell Signalling, Danvers, MA, USA), anti-FGFR-2 (#MAB684, R&D systems), anti-EGFR (#ab231, Abcam, Cambrigde, UK), anti-ErbB2/PE (#FAB6744, R&D systems), anti-ErbB3/APC (#FAB3481, R&D systems) and anti-ErbB4 (#MAB11311, R&D systems)**.** Primary VEGFR-1, VEGFR-2, FGFR-2 and ErbB4 antibodies were visualized using rabbit anti-mouse PE-conjugated secondary antibody (Dako Cytomation, Glostrup, Denmark) and FGFR-1 using FITC-conjugated swine anti-rabbit secondary antibody (Dako Cytomation). Primary EGFR was visualized using Alexa Fluor 488 conjugated anti-rat secondary antibody and PDGFRα was visualized using streptavidin/FITC (BD Biosciences). IgG-FITC/PE/APC and secondary antibodies were used as negative (isotype) controls. Expression was analyzed using FACScalibur1 (BD CellQuest Pro software, BD Bioscience). The data were analyzed using FlowJo software (Tree Star Inc., Ashland, Oregon, USA). Expression levels were considered as actual membrane protein expression when the percentage of RTK expressing cells reached 5% above its isotype control.

### Cell count and viability assays

Quantification of medulloblastoma cell count and cell viability was performed using crystal violet staining and WST-1 colorimetric viability assays. Cells were seeded in sextuple in a 96-well plate at a density of 15 x 10^3^ cells/well in DMEM/F12 containing 1% FCS. After adhesion cells were treated with crizotinib (0–9 μM) or canertinib (0–9 μM). After 1 hour of inhibitor treatment, cells were stimulated with growth factors (all 100ng/mL). To determine the single effect of growth factors on tumor cell count after 48h, cells were fixed with 8% formaldehyde for 20 minutes. Cells were washed followed by incubation with 0.04% crystal violet in 4% ethanol for 30 minutes. Cells were then washed, air dried and incubated with 1% SDS solution on a shaker for 1h. Optical density was measured at 595nm with a microplate reader (Bio-Rad, Hercules, CA, USA) and tumor cell count was calculated using an internal calibrator. To examine the effects of RTK inhibitors on tumor cell viability with or without growth factor stimulation, WST-1 colorimetric viability assays were used following manufacturer’s (Roche) protocol guidelines. Optical density was measured after 48h using a microplate reader at 450 nm (Bio-Rad, Hercules, CA, USA). Optical densities were subtracted by a blank control and were normalized in percentages relative to control-treated cells (100%). Effects of growth factors on tumor cell viability were plotted against inhibitor concentrations. Area Under the Curve (AUC) was calculated between inhibitor-treated cells with the addition of growth factor (AUC_inh+gf_) relative to inhibitor-treated cells only (100%) (AUC_inh_). Bypass of the effect of the RTK inhibitor on tumor cell viability was defined by the following equation:

$$\left(\left({AUC}_{inh+gf-}\;{AUC}_{inh+gf\;stdev}\right)-\left({AUC}_{inh}+{AUC}_{inh\;stdev}\right)\right)≻0$$

AUC_inh + gf_ = AUC of cells treated with inhibitor and growth factor

AUC _inh + gf stdev_ = Standard deviation of the AUC of cells treated with inhibitor and growth factor

AUC_inh_ = AUC of cells treated with inhibitor alone

AUC_inh stdev_ = Standard deviation of the AUC of cells treated with inhibitor alone

### Western blot analyses

To examine protein levels, 1 x 10^6^ cells were plated in T25 flasks in 5 mL DMEM/F12 containing 1% FCS. After adhesion cells were treated with optimal dosages of crizotinib (9 μM) or canertinib (9 μM) based on cell viability assays using different concentrations of inhibitor. Combined treatment consisted of crizotinib (9 μM) + canertinib (4 μM) or canertinib (9 μM) + crizotinib (4 μM). To detect RTK phosphorylation, different concentrations of growth factor (1–100ng/mL HGF or EGF) were added for 5 minutes after 1h of inhibitor treatment. For the detection of downstream signaling effectors, growth factor (100ng/mL HGF or EGF) was added for 1h after 1h of inhibitor treatment. Cells were lysed in laemmli sample buffer (Bio-rad laboratories). Proteins were separated by sodium-dodecyl sulfate–polyacrylamide gel electrophoresis (SDS-PAGE), and transported onto nitrocellulose membranes. Membranes were incubated overnight using monoclonal antibodies (dilution: 1:1000) for phospo-Erk (pERK1/2)(#9101), total ERK1/2 (tERK1/2)(#9102), phospho-Akt (S473) (pAkt)(#9271), total Akt (tAkt)(#9272), phospo-MET (pMET)(#3126), total MET (tMET)(#4560), phospho-EGFR (pEGFR)(#2234) and total EGFR (tEGFR)(#2232). β-actin (dilution: 1:10000) (#sc-47778, Santa Cruz Biotechnology, Heidelberg, Germany) was used as a loading control. Membranes were then incubated for 1 hour using HRP-conjugated secondary antibodies (Dako Cytomation). Proteins were visualized by chemiluminescence, on x-ray film, and scanned. Immunoblots were quantified using ImageStudio™ Lite software (Li-Cor Biosciences, Nebraska, USA)

### Antibody array kits

Human phospho-kinase antibody arrays (R&D Systems) and human growth factor antibody arrays (Abcam) included membranes containing multiple protein antibodies printed in duplicates to measure protein expression levels. 4 x 10^6 cells were plated in T75 flasks in 10 mL DMEM/F12 containing 1% FCS. After adhesion cells were stimulated with growth factor (100ng/mL HGF or EGF) for 1h, treated with crizotinib or canertinib for 1 day or lysed. All lysates were prepared according to manufacturer’s protocols and sample protein concentrations were determined by a bicinchoninic acid (BCA)-protein quantification assay (Pierce). The protein intensity spots were visualized by chemiluminescence, on an x-ray film, and scanned. Signal intensities of proteins were analyzed with array software (ScanAlyze, Eisen Software, http://rana.lbl.gov/eisen). Data normalization was conducted by subtraction of the mean array background intensity from the protein signal intensities of each individual spot. For the human phospho-kinase antibody kit, mean signal intensities of individual proteins from growth factor-treated cells were then subtracted by the mean signal intensities of individual proteins from the control cells. For the human growth factor antibody array kit, mean signal intensities of individual proteins were normalized to a control.

### MET knockdown experiment

RES256 and UW473 cells were stably transduced with mCherry coupled pLKO.1 Mission shRNA vectors (Sigma-Aldrich, Zwijndrecht, the Netherlands) against MET (shMET) using Fugene HD Transfection Reagent (Roche, Woerden, the Netherlands) according to the manufacturer’s instructions. A scrambled vector (shSCR) was used as a vector control. Four days after transduction, transduction efficiency and knockdown was confirmed by measuring mCherry and MET cell surface expression levels by means of flow cytometry analysis. On the same day (Day 4), cells were seeded in sextuple in a 96-well plate at a density of 2 x 10^3^ cells/well in DMEM/F12 containing 5% FCS. Cells were counted on day 5, 7, 9 and 11, using crystal violet staining as described above.

### Statistics

Effects of growth factors on tumor cell count and quantifications of western blots were compared using a student’s t-test. Different conditions in cell viability assays and western blots were compared, by grouping all medulloblastoma cell lines. Subsequently, a nonparametric wilcoxon matched-pairs signed ranks test was used for inter-group comparison between conditions. SPSS20 was used for statistical analysis and a confidence level of p < 0.05 was considered to be significant.

## Results

### MET and EGFR are highly expressed on cell surface of medulloblastoma cell lines

**Fig 1:**
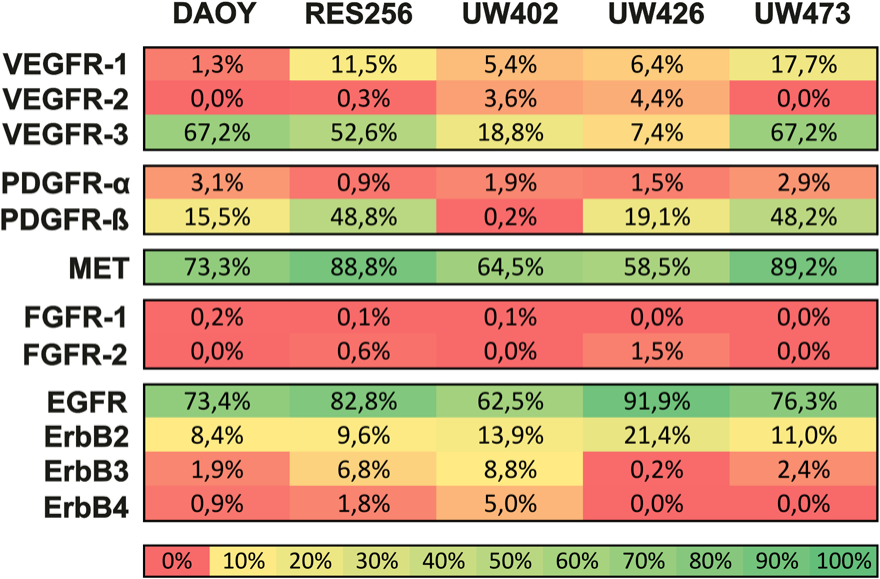
Cell surface RTK expression levels in medulloblastoma cell lines. Heatmap showing the cell surface expression levels of various RTK’s in percentages using flow cytometry analyses in medulloblastoma cell lines. Viable cells were stained with fluorescently labeled RTK-specific antibodies or isotype control antibody. Numbers represent the percentage of RTK-expressing cells, reaching above their isotype controls.

Important growth factors that are present in the central nervous system include VEGF, PDGF, HGF, FGF and EGF[9–13]. We expected that these growth factors influence cell signaling by binding to their RTK’s on the tumor cell surface leading to tumor cell proliferation. Therefore, we started to screen the expression of VEGFR1-3, PDGFRα/β, MET, FGFR1-2 and ErbB1-4 on the cell surface of 5 medulloblastoma cell lines (Fig 1 and S1 Fig). Flow cytometry analysis revealed that MET (mean±sd: 74.9% ± 13.9%), EGFR (mean±sd: 77.4% ± 10.9%) and ErbB2 (mean±sd: 67.8% ± 10.3%) are highly expressed in all medulloblastoma cell lines, whereas ErbB3 (mean±sd: 4.0% ± 3.6%) and ErbB4 (mean±sd: 1.5% ± 2.1%) are barely expressed at all (Fig 1 and S1 Fig). Variable expression was observed for VEGFR1, VEGFR3 and PDGFRβ, whereas VEGFR2, PDGFRα, FGFR1, FGFR2 were barely expressed at all (Fig 1). Since ErbB2 is unable to bind any known ligand and needs heterodimerization with other ErbB receptor family members (EGFR, ErbB3, ErbB4) for activation of its intracellular kinase domain, we further focused on the highly expressed and clinically relevant MET and EGFR receptors.

### HGF and EGF phosphorylate MET and EGFR, respectively

**Fig 2:**
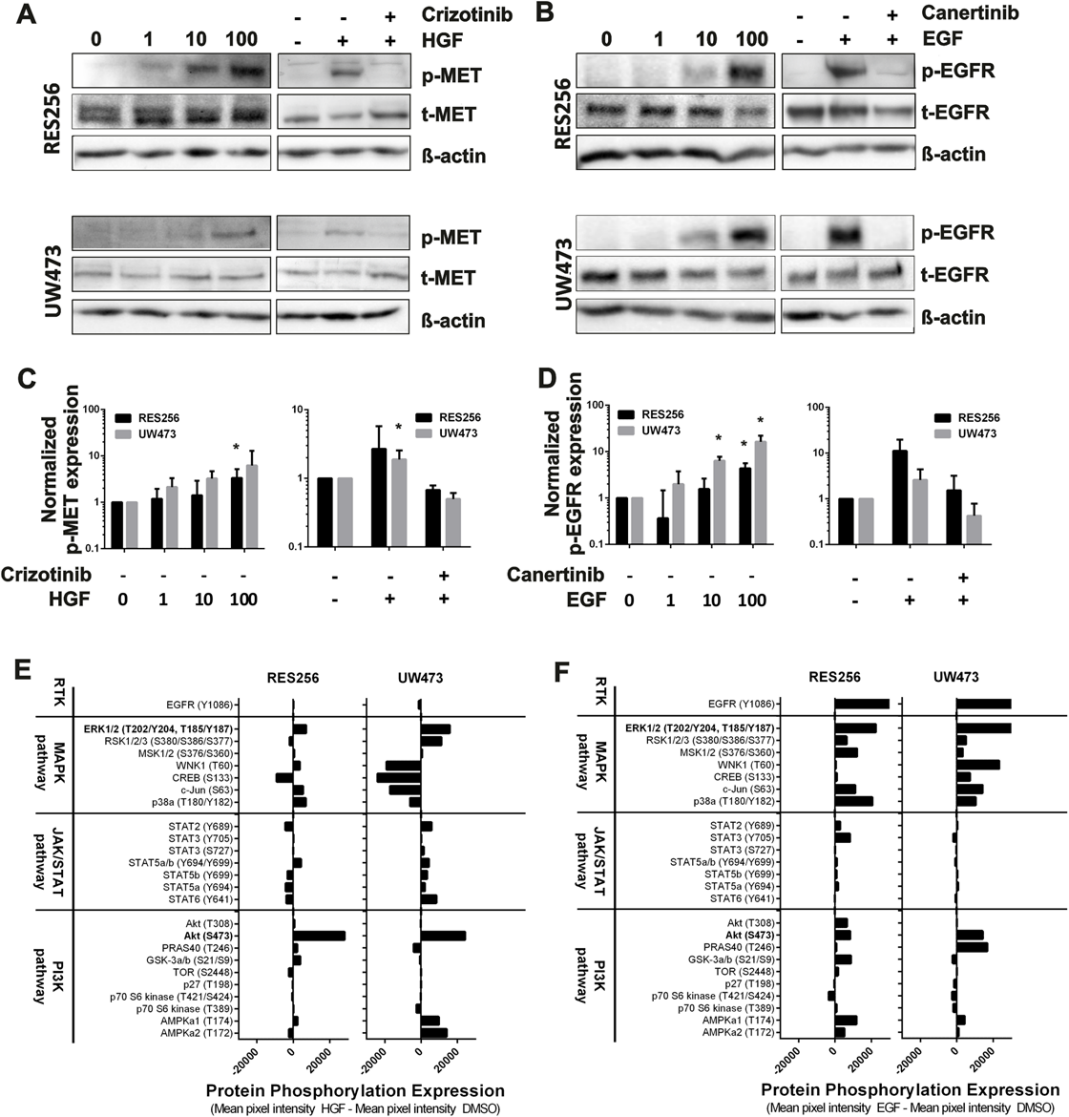
HGF and EGF phosphorylate MET and EGFR, respectively. **A-B** Immunoblots, showing the effects of **A** HGF stimulation (0-100ng/ml) in combination with crizotinib on MET (MET: 145kDa, pro-MET: 170kDa) phosphorylation and **B** EGF stimulation (0-100ng/ml) in combination with canertinib on EGFR (175kDa) phosphorylation in cell lines RES256 and UW473. Bands of MET represent MET (145kDa) and pro-MET (170kDa). Total-MET and total-EGFR were used as loading controls. **C-D** Quantification analysis of immunoblots showing the **C** normalized phospho-MET expression and **D** normalized phospho-EGFR expression. Quantitations are representative of 2 to 3 independent experiments. Student’s t-test: *p<0.05. **E-F** Quantitative analysis of the effects of **E** HGF and **F** EGF using human phospho-kinase arrays in cell lines RES256 and UW473. The plot displays the phosphorylation sites of the proteins present on the proteome array that are categorized upon different signal transduction pathways. Mean pixel intensities were calculated and subtracted by the mean background intensity of the array. Bars depict the difference between growth factor-stimulated cells and control cells. Positive values indicate growth factor-induced phosphorylation of the kinase.

Next, we examined the effects of exogenous growth factor stimulation and RTK inhibition on phosphorylation of MET and EGFR in cell lines RES256 and UW473. These cell lines were selected based on their high MET and EGFR cell surface expression compared to the other cell lines. Potential interesting RTK inhibitors that target MET or EGFR are crizotinib and canertinib, respectively. Stimulation with different concentrations of HGF (1–100ng/mL) resulted in the highest phosphorylation of MET at 100ng/mL in both cell lines (Fig 2A and 2C). When crizotinib was added to the cells, HGF stimulation resulted in a decreased phosphorylation of MET in both cell lines (Fig 2A and 2C). In the same way, EGF stimulation (1–100ng/mL) resulted in highest phosphorylation of EGFR in both cell lines at 100ng/mL, which was decreased when canertinib was added previously (Fig 2B and 2D). These results indicate that HGF and EGF phosphorylate their receptors MET and EGFR at 100ng/mL, which can be blocked with the RTK inhibitors crizotinib and canertinib, respectively. Based upon these results, we continued these experiment by using consequent identical levels of HGF and EGF (both 100ng/mL).

Downstream signaling proteins activated by HGF, included ERK1/2, c-Jun, p38a and Akt (S473). A decrease in phospho-protein expression was found for CREB in RES256 cells (Fig 2E and S2 Fig). In cell line UW473, a HGF induced increase in phospho-protein expression of ERK1/2, RSK1/2/3, Akt (S473), AMPKA1 and AMPKA2. Downregulated phospho-proteins in UW473 cells included WNK1, CREB, c-Jun, p38a and PRAS40 (Fig 2E, S2 Fig and S3 Fig). Stimulation with EGF increased phospho-protein expression of EGFR, ERK1/2, RSK1/2/3, MSK1/2, c-Jun, p38a, STAT3 (Y705), Akt (T308), Akt (S473), GSK-3a/b, AMPKA1 and AMPKA2 in RES256 cells (Fig 2F, S2 Fig and S3 Fig). EGF stimulation of UW473 cells increased phospho-protein expression of EGFR, ERK1/2, RSK1/2/3, WNK1, CREB, c-Jun, p38a, Akt (S473) and PRAS40 (Fig 2F, S2 Fig and S3 Fig). Altogether, these results indicate that EGF and HGF commonly activate critical downstream signaling effectors, including Akt and ERK1/2 signaling proteins.

### Exogenous HGF significantly bypasses the effect of ErbB inhibition on tumor cell viability in medulloblastoma cell lines

**Fig 3:**
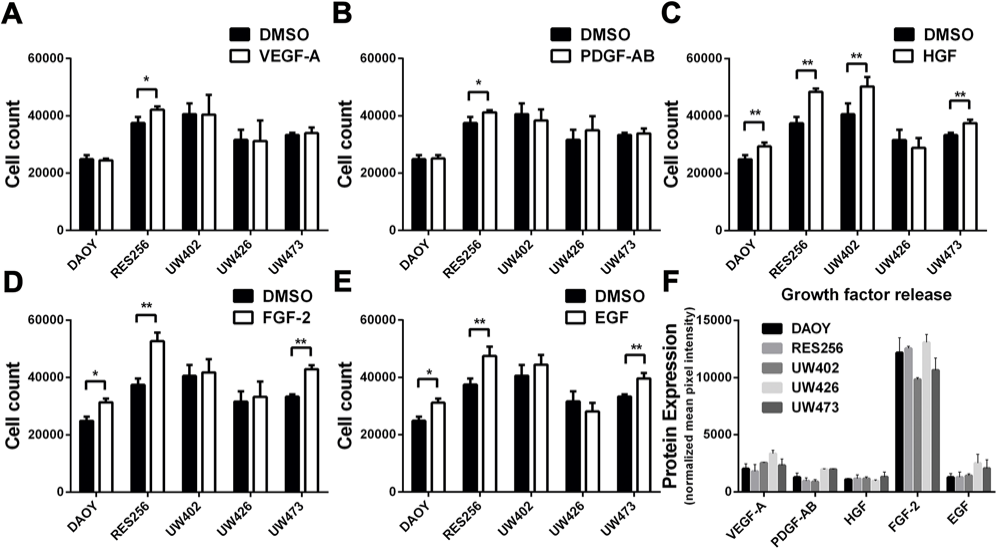
Effects of the addition of growth factors on tumor cell count in medulloblastoma cell lines. **A-E** Barplots, showing the effects of **A** VEGF-A, **B** PDGF-AB, **C** HGF, **D** FGF-2 and **E** EGF on cell count after 48h using crystal violet staining. Tumor cell count was calculated using an internal calibrator. Experiments were performed in sextuple. **F** Barplot, showing the protein expression levels of VEGF-A, PDGF-AB, HGF, FGF-2 basic and EGF in 5 medulloblastoma cell lines. Mean signal intensities of individual growth factors were normalized to a medium control. *p<0.05, **p<0.01

**Fig 4:**
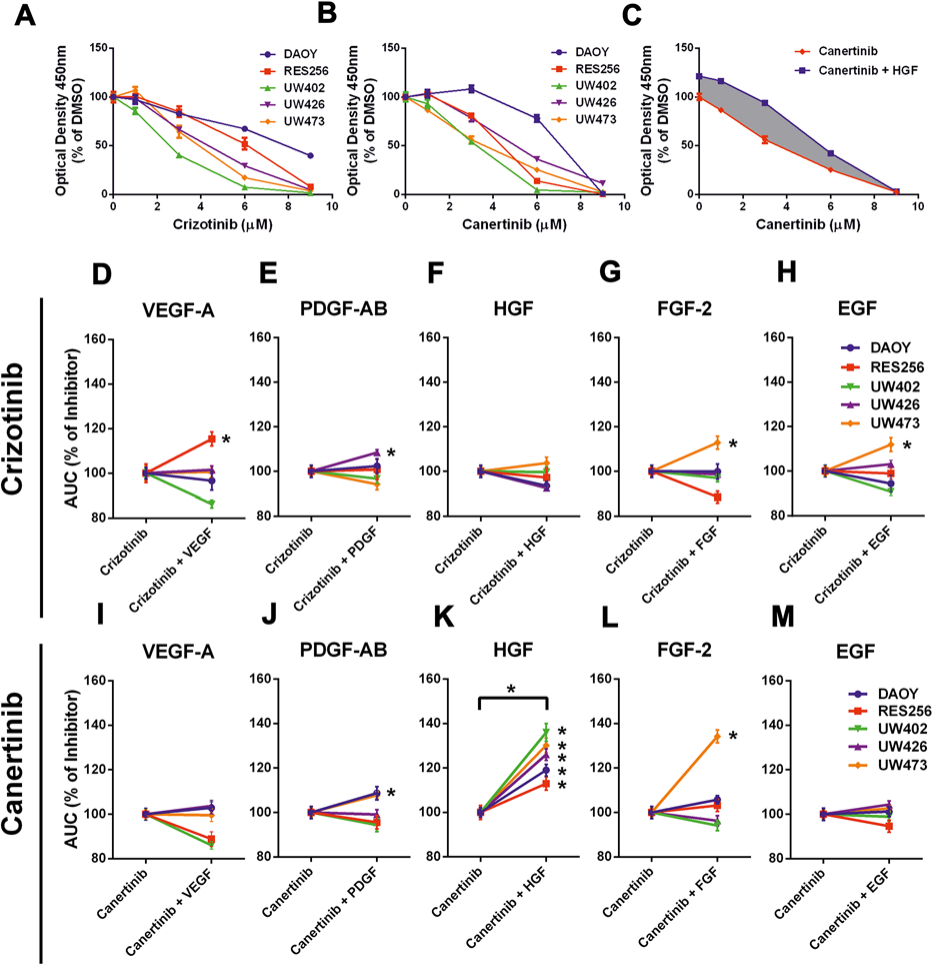
HGF significantly increases tumor cell viability in canertinib-treated medulloblastoma cell lines. Cell viability assays, showing the effects of different concentrations of **A** crizotinib and **B** canertinib on tumor cell viability in 5 medulloblastoma cell lines after 48h. **C** Cell viability assay, showing the effect of exogenous HGF in addition to canertinib on tumor cell viability in cell line UW473. The difference in area under the curve (AUC) is depicted by the grey area. **D-M** Cell viability assays showing the effects of exogenous VEGF-A, PDGF-AB, HGF, FGF-2 and EGF in addition to crizotinib or canertinib in medulloblastoma cell lines. Cells were stimulated with growth factor after 1h of RTK inhibition for 48 hours. Lines depict the relative AUC in percentages between inhibitor and growth factor-treated cells and inhibitor-treated cells only (100%). Asterisks indicate enhanced tumor cell viability and was defined by an increase in the relative AUC after subtraction of the standard deviations of all data points of both curves. A nonparametric wilcoxon matched-pairs signed rank test indicated significantly enhanced cell viability (*p<0,05) between HGF-stimulated cell lines compared to non-HGF stimulated medulloblastoma cell lines during canertinib treatment.

Tumors might circumvent the effects of kinase inhibitors due to the presence of microenvironmental RTK ligands, such as VEGF, PDGF, HGF, FGF and EGF. The single effect of stimulation with the above mentioned growth factors on tumor cell count are given in Fig 3A–3E. Medulloblastoma cell lines are most sensitive to exogenous HGF (4 out of 5 cell lines), FGF-2 (3 out of 5 cell lines) and EGF (3 out of 5 cell lines) resulting in increased tumor cell growth. Less sensitivity is observed for stimulation with VEGF-A and PDGF-AB. To investigate whether medulloblastoma cell lines release the above mentioned RTK ligands, we used a human growth factor antibody array kit. We observed that overall growth factor production was low in medulloblastoma cell lines, except for FGF-2 (Fig 3F). Since we did not detect FGFR1-2 cell surface expression, we continued our focus on MET and EGFR receptors. The single effects of crizotinib and canertinib on tumor cell viability are given in Fig 4A and 4B. We observed a dose-dependent decrease in tumor cell viability in all used medulloblastoma cell lines after treatment with either crizotinib (LC50 mean±sd: 5.0 μM ± 1.9 μM) or canertinib (LC50 mean±sd: 4.7 μM ± 1.5 μM).

The difference in tumor cell viability as a consequence of the addition of growth factors to a RTK inhibitor was calculated by the difference in area under the curve (AUC), depicted by the grey area in Fig 4C. The calculated differences in AUC for all growth factors used in combination with crizotinib or canertinib are shown in Fig 4D–4M. Upward lines marked with an asterisk indicate bypass of the effect of the RTK inhibitor on tumor cell viability by the addition of a growth factor. Whereas VEGF-A, PDGF-AB, FGF-2 and EGF show weak bypassing potential in addition to either crizotinib or canertinib, addition of HGF shows strong bypassing potential by significantly bypassing the growth-inhibitory effects of canertinib in medulloblastoma cells (Fig 4D–4M).

### Combined RTK inhibition blocks HGF-enhanced downstream signaling and tumor cell viability

**Fig 5:**
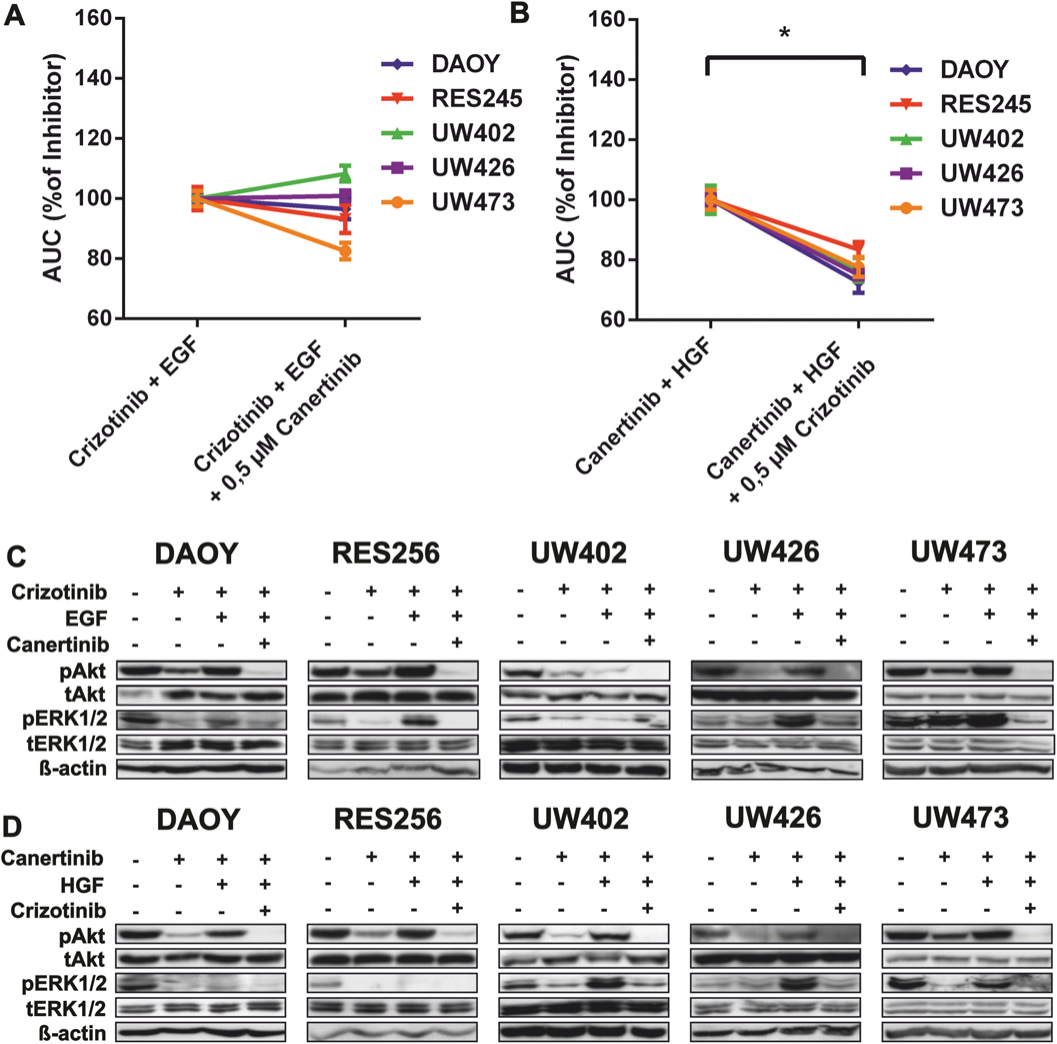
Combined RTK inhibition blocks HGF-enhanced downstream signaling and tumor cell viability. **A-B** Cell viability assays showing the effects of combined treatment with crizotinib and/or canertinib in medulloblastoma cell lines relative to single treatment in addition to **A** EGF or **B** HGF after 48 hours. Lines depict the percentage AUC of growth factor in addition combined RTK inhibition relative to growth factor in addition to single RTK inhibition (100%). A nonparametric wilcoxon matched-pairs signed rank test indicated significantly enhanced tumor cell viability (*p<0,05) between HGF-stimulated cell lines compared to non-HGF stimulated medulloblastoma cell lines in addition to canertinib. **C-D** Immunoblots showing the effects of **C** EGF and **D** HGF during single and combined treatment with crizotinib and/or canertinib on downstream signaling effectors Akt (S473) (60kDa) and ERK1/2 (44 kDa,42 kDa). β-actin was used as a loading control. Growth factors were added after 1 hour of inhibitor treatment. Lysates were made after 2 hours of inhibitor treatment.

We continued by combining specific RTK inhibitors to overcome the growth factor-induced bypass mechanism. Cell viability results demonstrated that addition of EGF to crizotinib showed no significant increase in cell viability and so no difference was seen when canertinib was added to decrease EGF-induced effects on cell survival (Fig 5A). In western blots, EGF addition showed increase of phospho-Akt and phospho-ERK1/2, whereas this effect was reversed by addition of canertinib (Fig 5C).

In Fig 5B, HGF in addition to canertinib demonstrated significantly increased cell viability in the cell cultures, whereas the HGF enhanced cell viability was reversed by the addition of crizotinib. Accordingly, western blots showed increased phospho-Akt and phospho-ERK1/2 upon HGF addition, whereas this effect was reversed by addition of crizotinib (Fig 5D).

**Fig 6:**
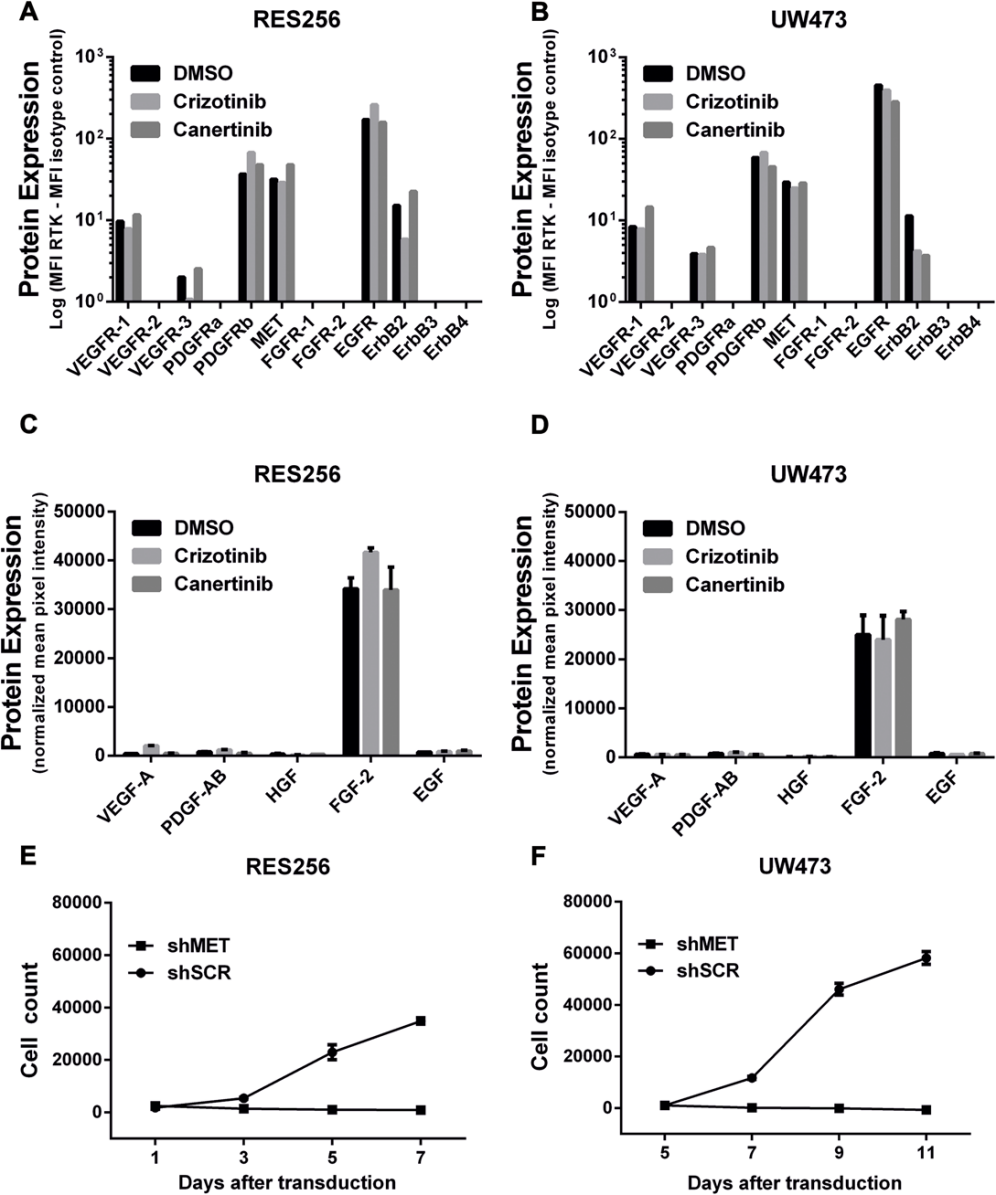
Crizotinib and canertinib does not affect RTK and growth factor expression levels in medulloblastoma cell lines. **A-B** Barplots, showing the cell surface expression levels of various RTK’s in MFI using flow cytometry analyses in **A** RES256 and **B** UW473 cells treated with crizotinib, canertinib or control. Viable cells were stained with fluorescently labeled RTK-specific antibodies or isotype control antibody. Log values represent the MFI of RTK-expressing cells, subtracted by the MFI of their isotype controls. **C-D** Barplots, showing the protein expression levels of VEGF-A, PDGF-AB, HGF, FGF-2 basic and EGF in **C** RES256 and **D** UW473 cell treated with crizotinib, canertinib or control. Mean signal intensities of individual growth factors were normalized to control. **E-F** Cell proliferation assays, showing the effect of MET knockdown on cell count in **E** RES256 and **F** UW473 cells using crystal violet staining. Cells were seeded at day 4 after transduction for 7 days at a density of 2 x 10^3^ cells/well in DMEM/F12 containing 5% FCS. Tumor cell count was calculated using an internal calibrator.

The above results suggest that exogenous HGF could be a key player in bypassing the effects of kinase inhibitors in medulloblastoma cells. To understand more about the mechanism of HGF/MET-induced bypass, we investigated the expression of the previously analyzed set of RTK’s on the cell surface of RES256 and UW473 cells upon treatment with crizotinib or canertinib. Treatment with crizotinib or canertinib had no effect on cell surface RTK expression levels compared to control-treated cells (Fig 6A and 6B). Moreover, we analyzed growth factor production in the absence or presence of crizotinib or canertinib and could conclude that the growth factor production in RES256 and UW473 cells did not differ upon addition of crizotinib or canertinib (Fig 6C and 6D). Since crizotinib is a dual inhibitor of MET and ALK, we used shRNA’s targeting MET to knockdown MET gene expression to be sure the crizotinib-induced decrease in cell viability was due to a blockade of MET. We could effectively knockdown MET in RES256 and UW473 cells using shRNA’s targeting MET (S4 Fig). Knockdown of MET resulted in decreased cell viability (Fig 6E and 6F), suggesting that MET is critical in the growth and survival of medulloblastoma cells. Altogether, these data indicate that the availability of exogenous HGF is of key importance in bypassing the growth-inhibitory effects of RTK inhibitors through activation of highly expressed MET

## Discussion

The present study demonstrates that exogenous HGF can significantly bypass the growth-inhibitory effects of canertinib in medulloblastoma cell lines. Previously with kinome profiling, we observed MET and EGFR/ErbB2 peptide activity in primary medulloblastoma samples[14]. In this study, RTK inhibitors crizotinib and canertinib were used to inhibit these targets, respectively. Tumor cell viability and downstream signaling were inhibited upon the single use of these inhibitors. The present study investigated the bypass potential of ligands commonly expressed in the brain tumor’s micro-environment.

MET was expressed on the cell surface of our medulloblastoma cell lines. This result is consistent with previous studies in medulloblastoma cell lines and primary tissue[15,27]. Furthermore, we show that EGFR and ErbB2 are highly and ErbB3 and ErbB4 are hardly expressed on the cell surface of our medulloblastoma cell lines. Expression of EGFR and ErbB2 is in agreement with previous studies in medulloblastoma cell lines and primary tissue expression, in which expression of EGFR and ErbB2 was detected in varying percentages[28]. More interestingly, high expression levels of both MET and ErbB2 in primary medulloblastoma tissue are correlated with poor clinical outcome in patients[15,16]. Therefore, MET and ErbB family receptors are interesting targets for RTK inhibitors in the treatment of medulloblastoma patients.

The anti-proliferative effects of crizotinib in our study underscores previous reports analyzing the anti-proliferative effects of MET TK inhibitors in medulloblastoma cell lines and mouse xenograft models[27,29,30,31]. Crizotinib is currently being assessed in pediatric clinical trials including medulloblastoma (NCT00939770). Although investigation of anti-tumor activity is still in progress, crizotinib was well tolerated in medulloblastoma patients[32]. In addition, administration of canertinib effectively inhibited cell viability in our medulloblastoma cell lines. These results are compatible with a previous study analyzing the effects of gefitinib (EGFR TK inhibitor) in medulloblastoma cell lines and xenografts[33]. To investigate whether ErbB family receptor inhibition could improve the clinical outcome of brain tumor patients, the use of single EGFR/ErbB2 TK inhibitors was analyzed for its anti-tumor activity in various clinical trials. EGFR/ErbB2 TK inhibitors were well tolerated, but more importantly, showed limited clinical response in brain tumor patients including patients with medulloblastoma (NCT00095940; NCT00077454)[17,18]. The contrasting results of pre-clinical medulloblastoma models and clinical trials might be explained by the fact that pre-clinical in vitro models often lack the natural tumor environment.

An emerging concept is the role of the tumor microenvironment in bypassing targeted therapies by producing ligands that can compensate for the drug-inhibited TK by phosphorylating alternative RTK’s. Growth factors, such as VEGF, PDGF, HGF, FGF and EGF are normally expressed during brain development. High expression levels of these growth factors were previously found in brain tumor bulk [9–13]. It is important to take into consideration that these results of crude tissue might have no relation with growth factors present on the cellular level, where mechanisms of bound and soluble growth factors play a crucial role in relation to specific stromal crosstalk. A limitation of the present study is that a certain amount of growth factor was added, whereas the exact amount present for interference with RTKs on a cellular level is still unknown. We are not aware of any possibility or study measuring exact growth factor availability on the cellular level. Therefore, different concentrations of growth factor were used to define the optimal dose for RTK phosphorylation in medulloblastoma cell lines based upon previous studies[22,23,34].

In our study, addition of the above mentioned growth factors to crizotinib or canertinib highlights the strong bypassing potential of HGF in addition to canertinib on medulloblastoma cell viability and downstream signaling effectors Akt and ERK1/2. Addition of EGF to crizotinib also resulted in increased Akt and ERK1/2 signaling, whereas it did not functionally affect the cell viability. These results suggest that other signaling proteins will be involved and counter balance the increased Akt and ERK1/2 signaling without resulting in altered cell viability. The HGF-induced bypass of canertinib was completely reversed by co-administration of MET inhibitor crizotinib, indicating that HGF was acting through its receptor MET. This was underscored by the finding that targeting MET using shRNA, results in decreased cell viability in medulloblastoma cell lines. The phenomenon of HGF/MET-induced bypass is in line with previous reports in other tumor types. A substantial increase in HGF and/or MET expression levels as a consequence of EGFR inhibition was shown to be responsible for the bypass of RTK inhibition in adult glioblastoma and breast cancer[20,35]. In our study, the addition of RTK inhibitors did not increase HGF or MET expression levels. Unlike in our medulloblastoma cell lines, HGF is constantly produced *in vivo*[14,36], suggesting potential bypass mechanisms related to the tumor micro-environment.

In conclusion, this manuscript describes the bypassing capacity of paracrine HGF in medulloblastoma cell lines. MET and EGFR were highly activated in primary medulloblastoma samples and subsequently high percentages of medulloblastoma cells expressed these RTK’s. Our study highlights the extensive importance of HGF present in the tumor micro-environment in bypassing the effects of RTK inhibitors. These findings provide a rational explanation for the limited efficacy of single RTK inhibitors in medulloblastoma clinical trials. Moreover, the successful combination treatment to overcome this bypass mechanism supports the importance of combination therapies in medulloblastoma. Further studies in vivo using animal models will be warranted to support this hypothesis. Anticipating on these results by the development of novel (pre-) clinical trials with specific TK inhibitors in combination with a MET inhibitor seems very important to improve the clinical efficacy of novel targeted therapies in medulloblastoma in the future.

## Supporting Information

S1 Fig MET and EGFR are highly expressed on the cell surface of medulloblastoma cell lines. Results of flow cytometry analysis, showing histograms of MET, EGFR, ErbB2, ErbB3 and ErbB4 cell surface expression levels (blue) compared to their isotype controls (red) in medulloblastoma cell lines DAOY, RES256, UW402, UW426 and UW473. Viable cells were stained with anti-MET, anti-EGFR, anti-ErbB2, anti-ErbB3 or anti-ErbB4 antibodies and the percentage of RTK-expressing cells was calculated by subtraction of isotype controls.
(TIF)

S2 Fig Effects of MET knockdown on MET expression and cell proliferation. Phospho-kinase proteome array membranes of cell lines RES256 and UW473 showing the effects **A** HGF or **B** EGF stimulation on downstream signaling. Phospho-proteins that showed the most robust changes in phosphorylation are indicated by numbers that are explained underneath the membranes.
(TIF)

S3 Fig HGF and EGF stimulation results in Akt and ERK1/2 phosphorlyation. Western blots showing the effects of HGF (left panel) and EGF stimulation (right panel) on phosphorylation of critical downstream signaling effectors Akt and ERK1/2.
(TIF)

S4 Fig Effects of MET knockdown on MET expression and cell proliferation. **A-B** Results of flow cytometry analysis, showing dotplots and histograms of MET cell surface expression in shSCR (red) and shMET (blue) in cell lines **A** RES256 and **B** UW473.
(TIF)
