# Supplementary figures and images for: Exogenous HGF Bypasses the Effects of ErbB Inhibition on Tumor Cell Viability in Medulloblastoma Cell Lines
Source: PLoS One. 2015 Oct 23;10(10):e0141381. doi: 10.1371/journal.pone.0141381 (PMC4619778; doi:10.1371/journal.pone.0141381)

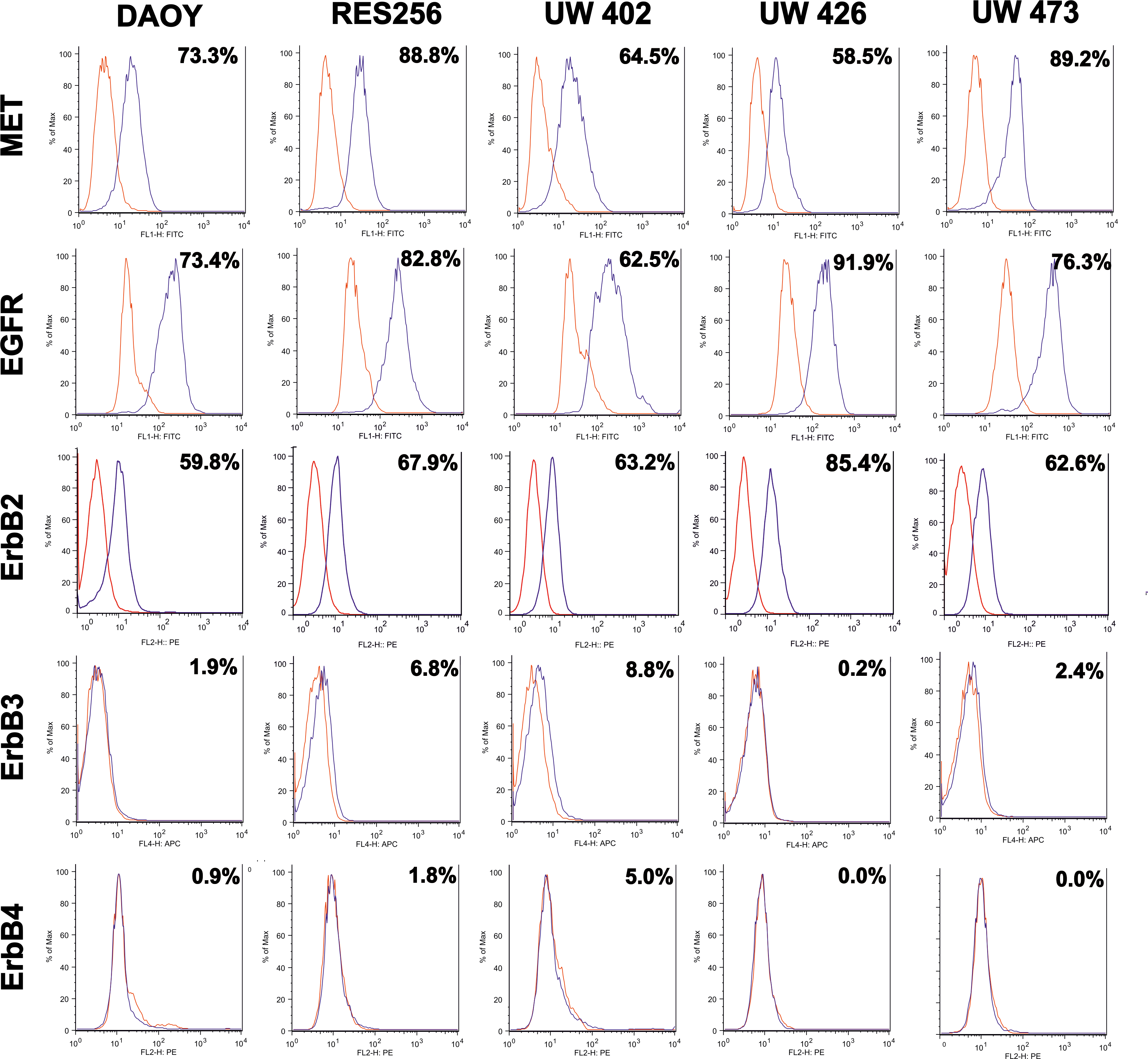

Supplement: S1 Fig — Results of flow cytometry analysis, showing histograms of MET, EGFR, ErbB2, ErbB3 and ErbB4 cell surface expression levels (blue) compared to their isotype controls (red) in medulloblastoma cell lines DAOY, RES256, UW402, UW426 and UW473. Viable cells were stained with anti-MET, anti-EGFR, anti-ErbB2, anti-ErbB3 or anti-ErbB4 antibodies and the percentage of RTK-expressing cells was calculated by subtraction of isotype controls. (TIF) [file pone.0141381.s001.tif]

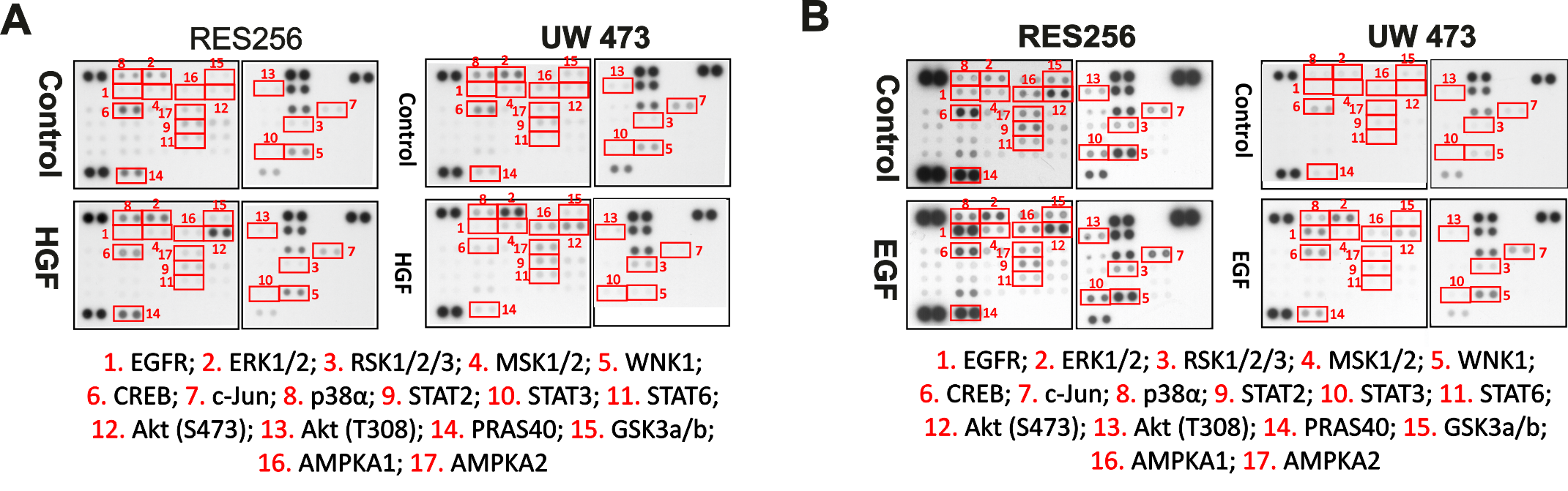

Supplement: S2 Fig — Phospho-kinase proteome array membranes of cell lines RES256 and UW473 showing the effects A HGF or B EGF stimulation on downstream signaling. Phospho-proteins that showed the most robust changes in phosphorylation are indicated by numbers that are explained underneath the membranes. (TIF) [file pone.0141381.s002.tif]

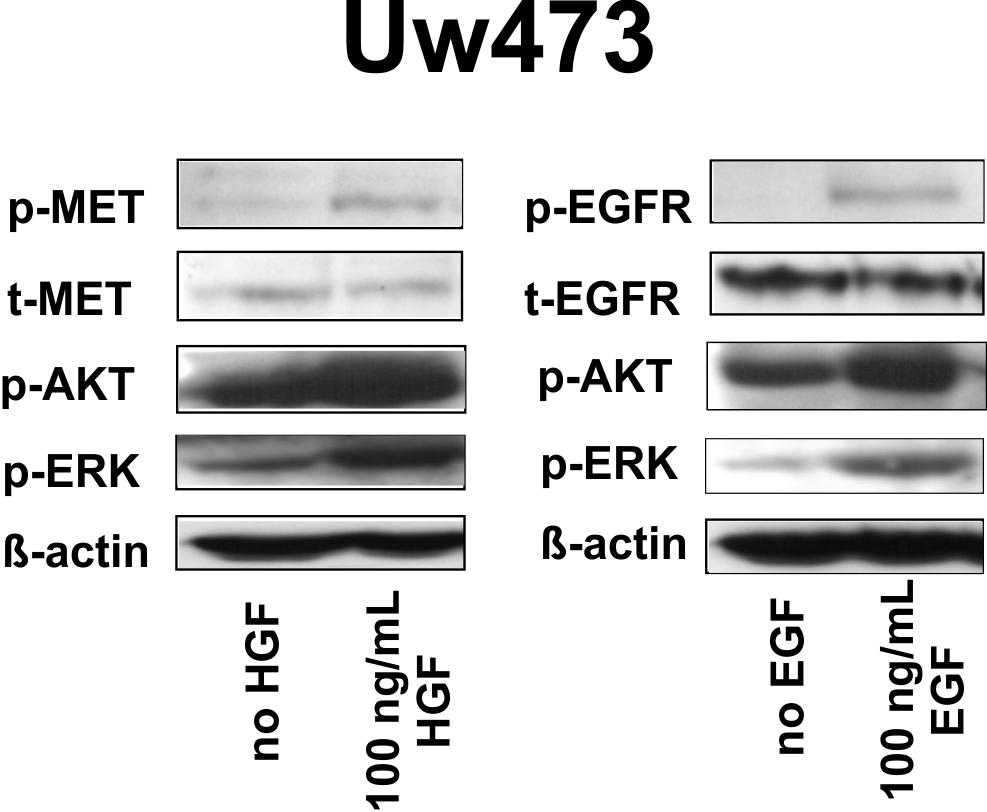

Supplement: S3 Fig — Western blots showing the effects of HGF (left panel) and EGF stimulation (right panel) on phosphorylation of critical downstream signaling effectors Akt and ERK1/2. (TIF) [file pone.0141381.s003.tif]
